# Survival Outcomes of Newly Diagnosed Multiple Myeloma at a Tertiary Care Center in North India (IMAGe: 001A Study)

**DOI:** 10.1200/GO.20.00625

**Published:** 2021-05-17

**Authors:** Uday Yanamandra, Rajni Sharma, Siddharth Shankar, Shikha Yadav, Rajan Kapoor, Suman Pramanik, Ankur Ahuja, Rajiv Kumar, Sanjeevan Sharma, Satyaranjan Das, Tathagata Chatterjee, Venkatesan Somasundaram, Tarun Verma, Kundan Mishra, Jasjit Singh, Ajay Sharma, Velu Nair

**Affiliations:** ^1^ Army Hospital (RR), Delhi, India; ^2^ IMAGe Research Scholar, Manipal Hospital, Delhi, India; ^3^ Command Hospital (EC), Kolkata, India; ^4^ Command Hospital (AF), Bengaluru, India; ^5^ Command Hospital (CC), Lucknow, India; ^6^ Command Hospital (SC), Pune, India; ^7^ Command Hospital (WC), Chandimandir, India

## Abstract

**PURPOSE:**

The outcomes of patients with myeloma from developing countries are often lacking because of poor record maintenance. Publications from such settings are also limited because of the retrospective nature of the data collection. Information technology can bridge these gaps in developing countries with real-time data maintenance. We present the real-time survival data of the patients with myeloma from a tertiary care center in North India using one such indigenously built software.

**PATIENTS AND METHODS:**

These are real-time data of all patients with myeloma presenting to a tertiary care center from North India. The patient characteristics (demographics, baseline disease characteristics, risk stratification, and outcomes) were recorded contemporaneously. The survival of the study population was analyzed and grouped based on various disease characteristics at diagnosis.

**RESULTS:**

The median age of the study population (N = 696) was 65.9 (34.9-94.9) years with male predominance (65%). The median follow-up was 3.7 years (0-18.6 years) with the median overall survival (OS) not achieved. The OS of the study population at 1, 3, and 5 years was 94% (n = 558), 87.5% (n = 394), and 83.1% (n = 267), respectively. Most of the patients presented in advanced stages based on International Staging System (III:70%). On Kaplan-Meier analysis, the presence of weight loss (*P* = .01), renal dysfunction (*P* = .047), and anemia at diagnosis (*P* = .004) had a significant impact on survival. On Cox proportional model univariate analysis, the presence of renal dysfunction, anemia, and weight loss had the significant hazard ratio of 1.68 (1-2.82, *P* = .049), 3.18 (1.39-7.29, *P* = .0063), and 2.81 (1.22-6.42, *P* = .014), respectively, whereas on multivariate analysis of hypercalcemia, renal disease, anemia, and bone disease (CRAB) features, only anemia was found to have a significant hazard ratio of 2.56 (1.01-6.47, *P* = .046).

**CONCLUSION:**

The real-world data show OS comparable with the published western literature. Only anemia was found to have significant impact on survival. The use of such software can aid in better data-keeping in resource-constrained settings.

## INTRODUCTION

Multiple myeloma (MM) is an incurable disease with relapsing-remitting nature. It is characterized by the neoplastic proliferation of clonal plasma cells producing excess monoclonal immunoglobulin, light chains, or both, often resulting in a multitude of target organ damage. Managing a patient with MM in India has several constraints, including the availability of infrastructure (equipment, expertise, and quality assurance), reluctance to avail autologous stem-cell transplant (ASCT), frequent change of health care facilities, and lack of data sharing between institutions and health insurance.^1,2^ Survival depends on multiple factors including type and duration of therapy, initial response, compliance to therapy, availability of drugs, and feasibility of providing ASCT.^3^ In India, the outcome in MM has improved substantially over recent years, as a result of the availability of multiple novel agents with an acceptable safety profile.^2^ Also, the increasing number of autologous transplants in the country has improved the overall survival (OS) of these patients.^4^ The overall impact on survival by improved strategies in the country has rarely been reported.^2^

**CONTEXT**

**Key Objective**
We present data from a single referral institution highlighting how a concerted effort and the use of patient-reported outcomes through a hybrid application can easily facilitate data collection and patient follow-up.
**Knowledge Generated**
The 5-year overall survival of the study population even in resource-constrained settings was comparable with western literature being 83%. We had more patients belonging to advanced International Staging System stages. Among all the clinical and demographic characteristics, on multivariate analysis, only anemia was found to have significant impact on survival. Our study followed up a large study population (n = 696) in real time using an online platform and hybrid application in a multiethnic clientele.
**Relevance**
The use of such hybrid applications can aid in better data-keeping in resource-constrained settings.

The studies on the impact of various baseline characteristics on the overall outcomes of patients with myeloma from real-world settings are lacking.^5,6^ Similarly, the relevance of the currently used prognostic staging (international staging system [ISS]) in real-world settings outside clinical trials is rarely studied. The primary aim is to study the survival outcomes of patients with newly diagnosed multiple myeloma (NDMM) and stratify the outcomes based on baseline disease characteristics and staging. We also evaluated the impact of the presence of weight loss, infections, or co-morbidities at the time of diagnosis on the OS of these patients.

## PATIENTS AND METHODS

This was an observational study of patients with NDMM managed at a tertiary care center from North India where data were contemporaneously entered in an online indigenously created platform^7^ from January 1, 2017, to July 31, 2020. The platform was created by the primary author and was supported by the Indian Myeloma Academic Groupe as the IMAGe-001 study, wherein the participating centers can enter the details independently without any sharing agreement. Patients who were diagnosed with MM based on International Myeloma Working Group guidelines and being followed up at our center were included in this study and the software.^8^ Written informed consent was obtained from all patients. The software (hybrid application) has a web-based component that is handled by the treating doctors (used for entering the sociodemographic features and therapy details) and a patient-operated mobile application component (used for entering their complaints or events). These details are entered on the platform in a real-time fashion. As an autofeedback mechanism, the treating team would call any patient who has not reported (either physically or on the mobile application) for three months. The treating team or the next-of-kin contemporaneously entered the outcomes (including any events) through the connected mobile application.

Clinical variables highlighted in this study included the presence of weight loss; co-morbidities; infections; and hypercalcemia, renal disease, anemia, and bone disease (CRAB features as per International Myeloma Working Group criteria)^8,9^ at diagnosis. The survival of the patient was assessed based on these outcomes. The ISS was used for the risk stratification of the disease.^10^ Patient outcome was assessed in terms of OS and was further grouped based on the abovementioned clinical variables. Institutional ethical clearance was obtained for collecting and analyzing the data.

JMP 15.0 was used for statistical analysis. The distribution of the variables is described as mean ± standard deviation unless specified otherwise. A *P* value ≤ .05 was considered significant. The survival of the patients was assessed using Kaplan-Meier survival analysis, and the difference between the curves analyzed using the log-rank test. Cox proportional hazard model was used for determining the hazard ratios (HRs).

## RESULTS

**FIG 1:**
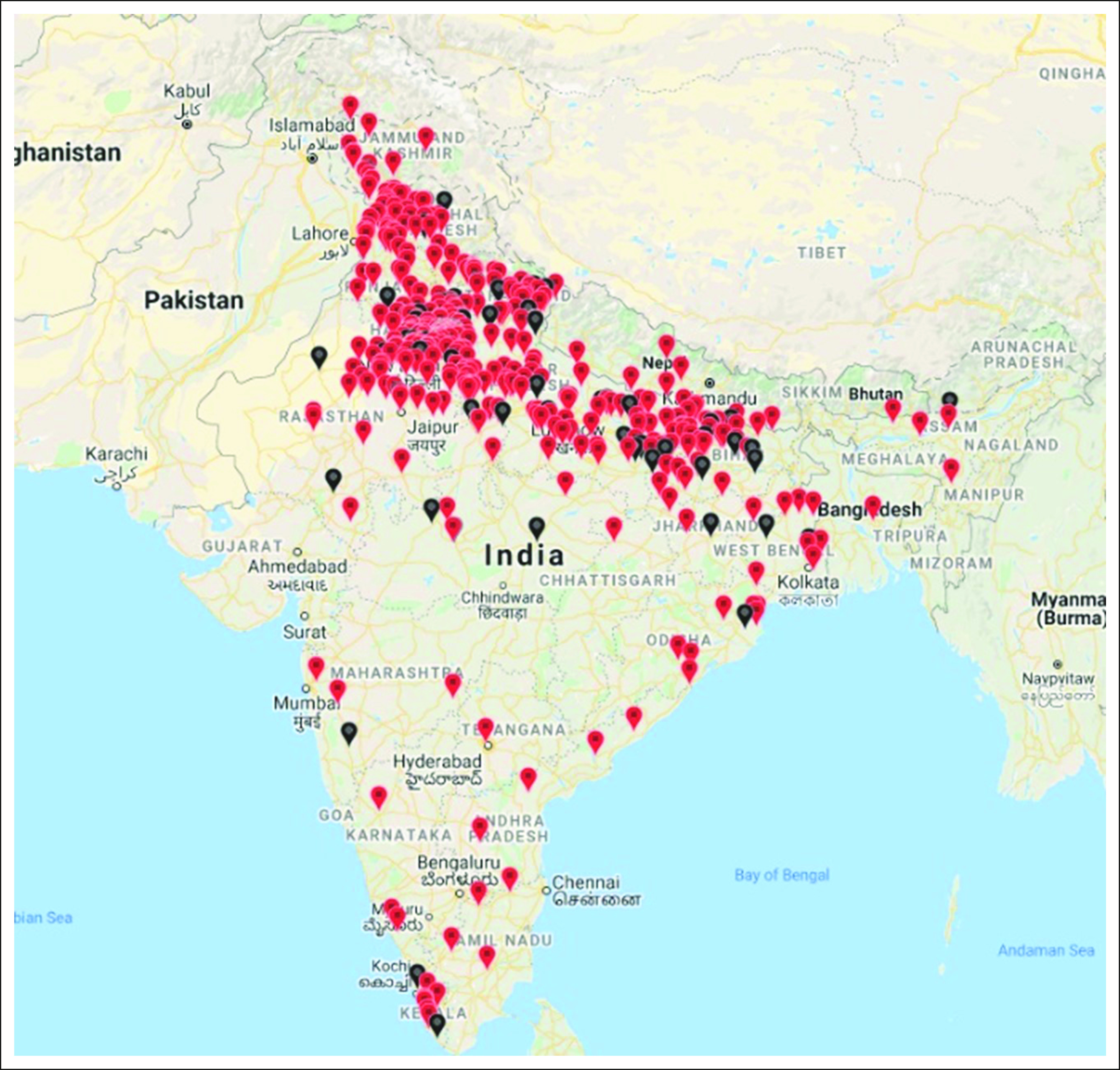
Geographical distribution of the study population (patients who have succumbed to the illness are depicted in black and those still surviving as red tags).

A total of 696 patients, 65% males, were enrolled in our study. The geographical distribution of the patients enrolled in the study is shown in Figure 1, and patient and disease characteristics are shown in Table 1. The cytogenetic data were available for 647 patients with the majority of patients (n = 599, 92.58%) having no reported cytogenetic abnormalities with mere 7.42% (n = 48) having cytogenetic abnormalities. Among those with positive results, more than half of the patients had del17p abnormalities followed by t(4;14); 58.33% and 18.75%, respectively.

**TABLE 1:**
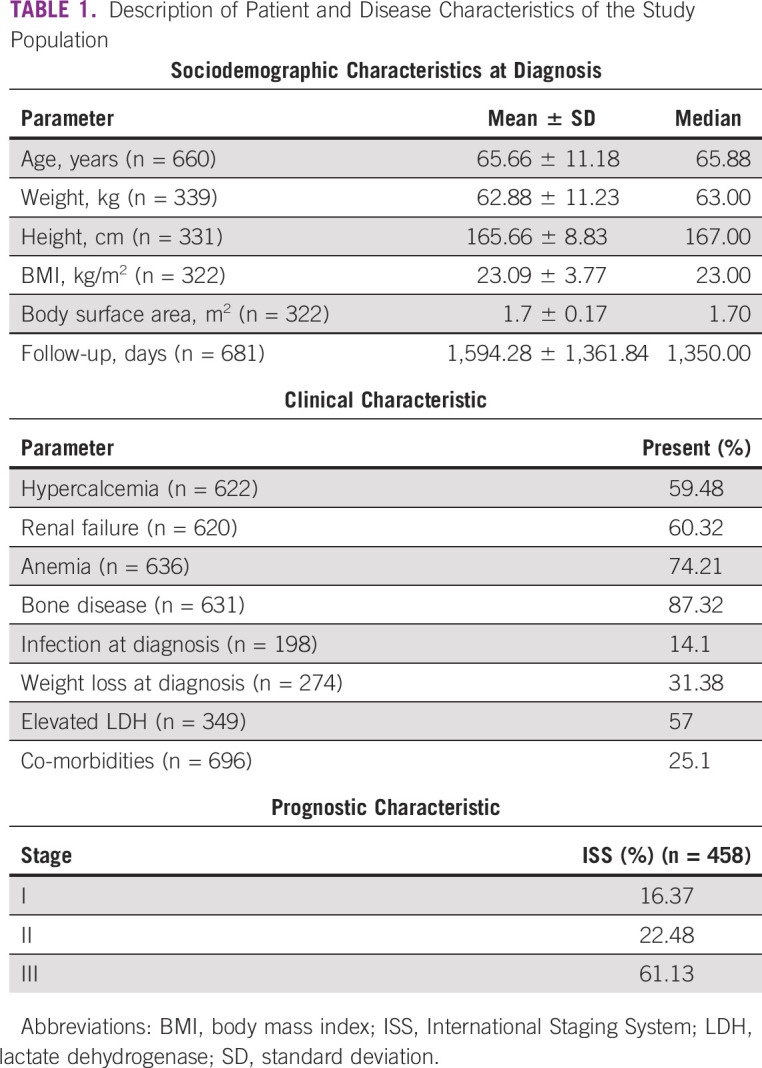
Description of Patient and Disease Characteristics of the Study Population

**FIG 2:**
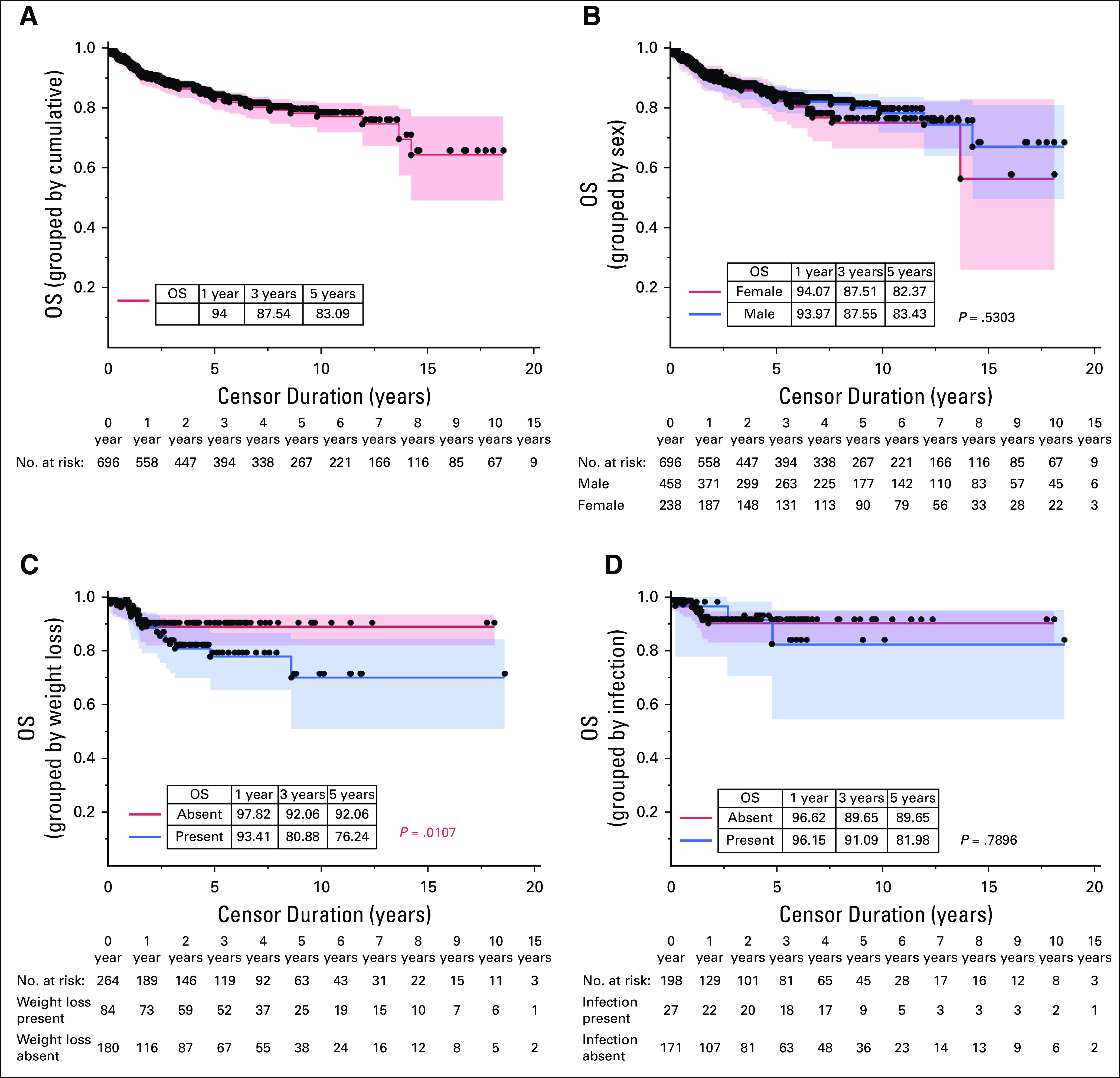
OS grouped by (A) cumulative, (B) sex, (C) weight loss, and (D) infection. OS, overall survival.

The median OS of the study population was not achieved. The OS of the patients at the end of 1 year, 3 years, and 5 years was 94% (n = 558), 87.5% (n = 394), and 83.1% (n = 267), respectively (Fig 2A). Kaplan-Meier analysis to compare the 1-year, 3-year, and 5-year OS grouped by various clinical and demographic variables is tabulated in Appendix Table A1. The HRs based on univariate analysis for the presence of CRAB features, weight loss, infections, female sex, and lactate dehydrogenase (LDH) at diagnosis were 1.47 (0.89-2.41, *P* = .13), 1.68 (1-2.82, *P* = .049), 3.18 (1.39-7.29, *P* = .0063), 0.61 (0.32-1.15, *P* = .13), 2.81 (1.22-6.42, *P* = .014), 1.19 (0.33-4.21, *P* = .79), 1.14 (0.76-1.71, *P* = .53), and 1.78 (0.95-3.32, *P* = .07), respectively. On univariate comparison of ISS stages I and II in comparison with stage III ISS, the HRs were 0.44 (0.16-1.22, *P* = .67) and 0.57 (0.28-1.16, *P* = .12), respectively. On multivariate analysis, only the presence of anemia was found to have a significant difference of 2.56 (1.01-6.47, *P* = .046).

**FIG 3:**
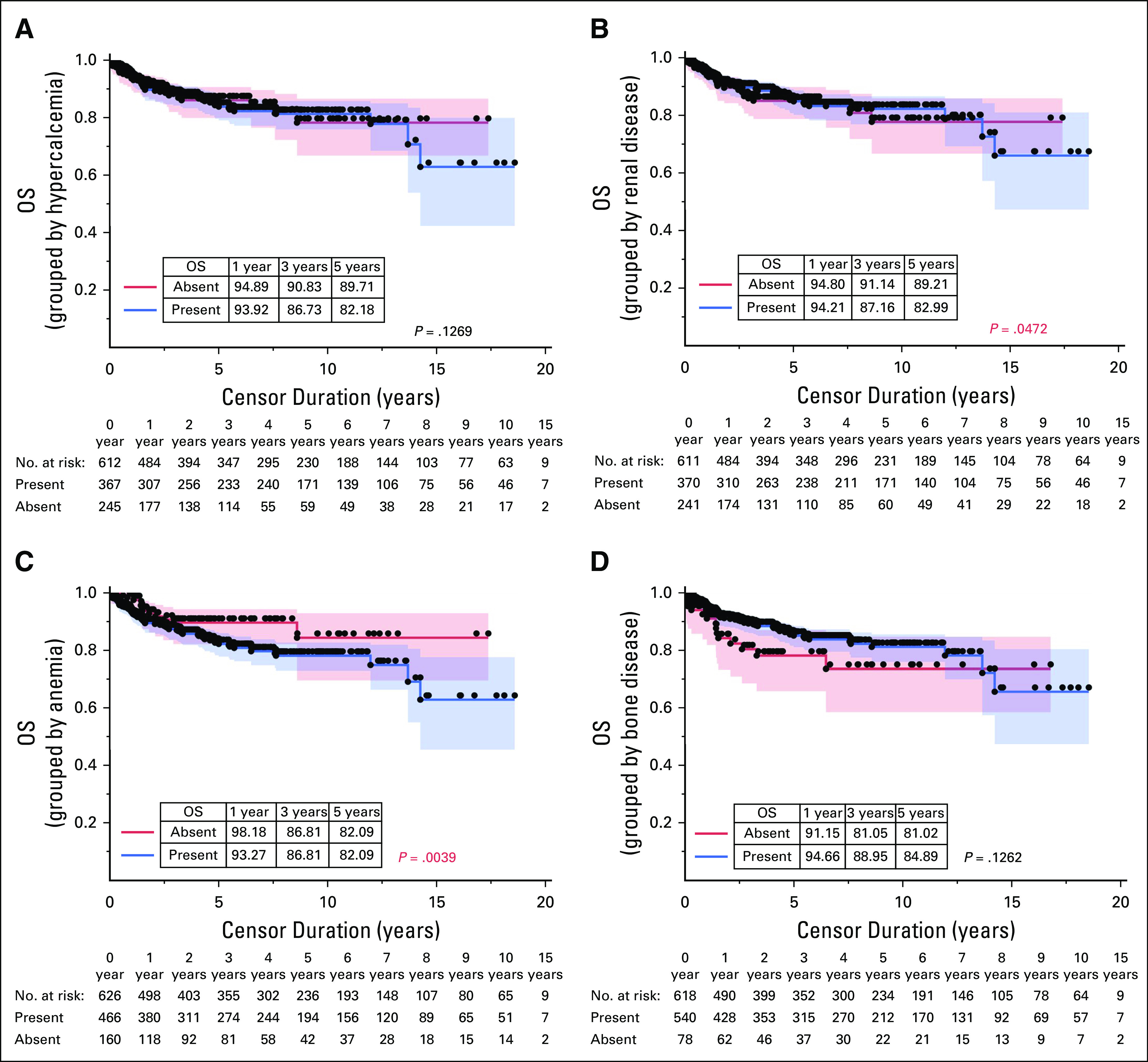
OS grouped by (A) hypercalcemia, (B) renal disease, (C) anemia, and (D) bone disease. OS, overall survival.

OS grouped by sex, presence of weight loss at diagnosis, and presence of infection at the time of diagnosis is illustrated in Figures 2B-2D. OS related to the various CRAB features is shown in Figures 3A-3D. The clinical variables whose presence was associated with inferior survival included weight loss at presentation (*P* = .01), renal failure (*P* = .047), and anemia (*P* = .004).

**FIG 4:**
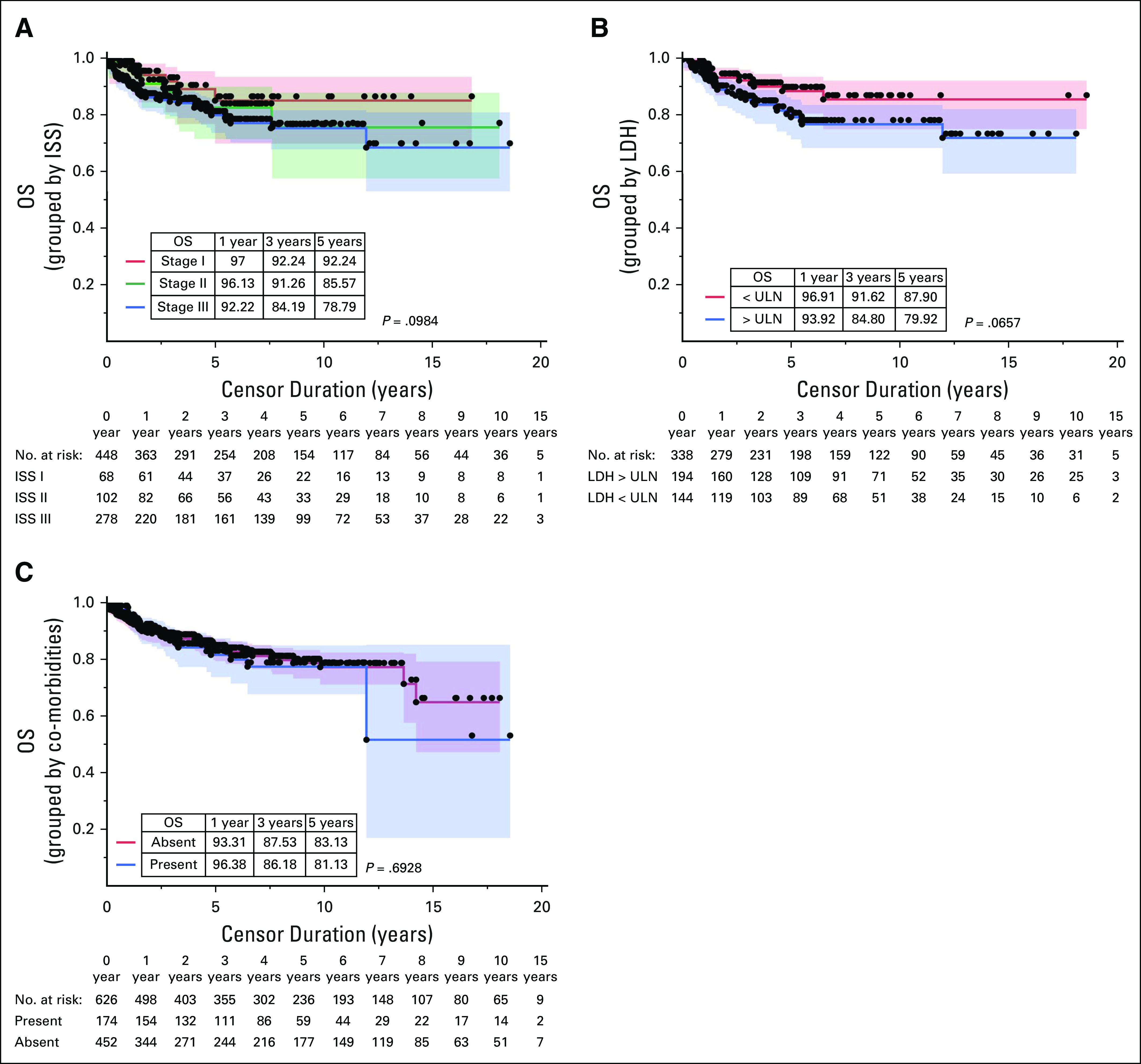
OS grouped by (A) ISS staging, (B) serum LDH level, and (C) co-morbidities. ISS, International Staging System; LDH, lactate dehydrogenase; OS, overall survival; ULN, upper limit of normal.

The study population was classified using ISS staging, and the distribution is given in Table 1. The presence of stage III disease by ISS was associated with reduced OS. Elevated serum LDH levels were also correlated with a lower OS; however, it was not statistically significant. OS has not shown any statistical difference when stratified by the ISS staging, LDH, and co-morbidities as illustrated in Figures 4A-4C.

## DISCUSSION

MM is the second most common hematological malignancy.^11^ The epidemiology and outcomes of the disease in India are often considered different from the west owing to an earlier age of onset and limited resources.^2,6^ However, most of these studies are limited by selection bias and their deductions based on secondary objectives. Our study included all sequential patients managed at a tertiary care center in Northern India to avoid these biases in prospectively reporting the epidemiology of MM from this part of the world. Myeloma is classically considered as a disease of the elderly, with a reported mean age of incidence ranging between 65 and 75 years of age in Western literature. Various studies from India have conventionally reported the mean age to be younger by a decade than the west, with individual studies even showing considerable adolescent and young adult MM.^12^ In our study, the mean age was on a par with that reported in the west. The age group was higher than that in other Indian studies.^12^

Several studies have reported the impact of the first-line therapy and short-term outcomes of MM. Since MM is a chronic disease, assessment of long-term survival is a crucial component for assessing disease biology. Treatment options for MM have seen a marked improvement in the past two decades, especially with the introduction of novel agents and increased availability of ASCT, leading to an improvement in OS and progression-free survival. The various disease characteristics as compared with other Indian and Western studies are elaborated in Appendix Table A2.^13-22^

Estimated OS in our study (Fig 2A) was higher than those reported in other studies (western and Indian) as illustrated in Table 2.^14,16,18,21,23,24^ The higher OS in our study can be attributed to a better hospital network, pan-India referral system, and fully sponsored therapy with no cost to the patient and better living standards of our clientele.

**TABLE 2:**
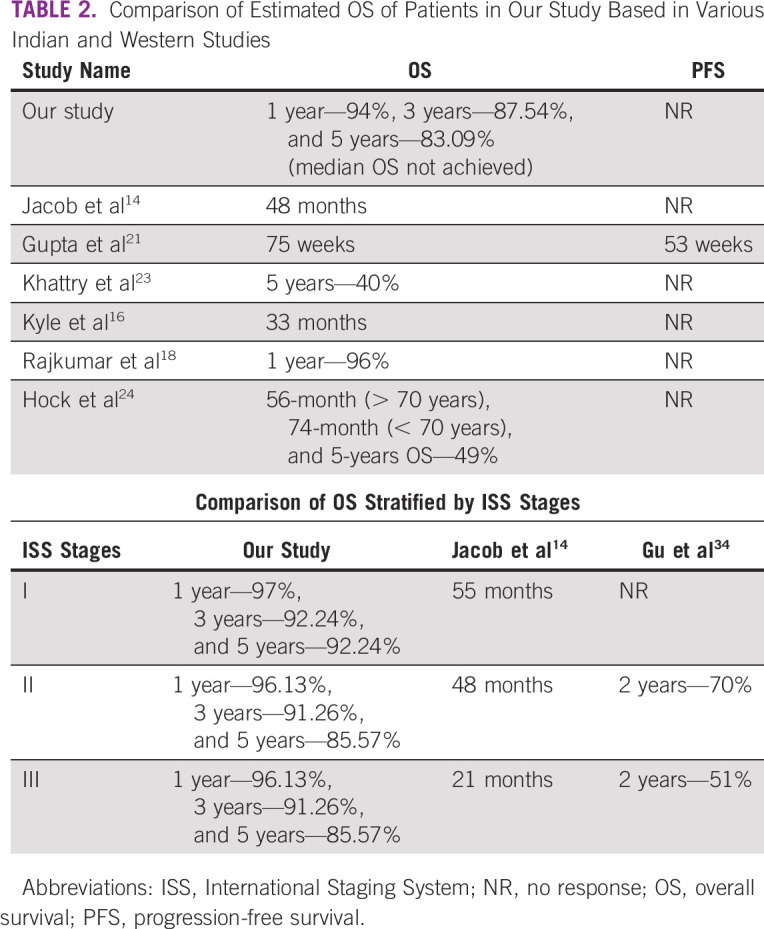
Comparison of Estimated OS of Patients in Our Study Based in Various Indian and Western Studies

Across various studies worldwide, males were found to have a higher predilection for MM, with the contrary rarely being reported. The sex affliction ratio in favor of males is widely variable, ranging from 1.3 to 2.1.^14,25,26^ Our study did not find any difference in survival between both sexes (Fig 2B), corresponding to western literature.^27^ On the contrary, the Chinese have demonstrated better OS in female vis-à-vis male (40 *v* 28 months, respectively).^28^

The incidence of weight loss was only 31% in the study population, coinciding with the western literature (24%) and contrary to the reported Indian data (75%).^15,17^ The incidence of weight loss was significantly less in the western population as compared with the Indian population, which can be attributed to lower per-capita income, leading to more impoverished living conditions, overcrowding, and malnutrition.^15,17^ These factors were mostly not applicable to our clientele owing to better standards of living and per-capita income. The median survival was significantly lower among patients with weight loss (21.09 months *v* 84.02 months, *P* = .01; Fig 2C) comparable with western literature (25.5 months).^29^ The significant impact of weight loss on the survival outcomes can be attributed to low body reserves, disease-related cachexia, advanced disease, and late presentation. Weight loss in these patients can result in hypoalbuminemia, which can further lead to higher plasma drug levels and subsequent drug-related adverse events.

Infection at diagnosis was found in 16%-23% in the published literature, which is higher than 14% seen in our study.^15,18,30^ The presence of infections led to poorer survival; however, the difference was not significantly different in our study (Fig 2D). Barila et al reported a median OS of 42 months among patients with infections compared with 54.36 months in our study.^31^ The presence of infections can be attributed to the immunoparesis in MM.^32^ In a Danish population-based study, immunoparesis did not have an impact on OS.^32^

Compared with other studies, an increased incidence of hypercalcemia,^14,16^ renal dysfunction,^14,16^ higher ISS staging,^14,33,34^ and increased LDH^21,22^ can be attributed to the delayed presentation to the health care facilities. Despite the provision of comprehensive health care services and a good network, patients are presenting late because of a lack of awareness of early symptoms and hesitation in consulting doctors. Moreover, in the online platform,^7^ CRAB features are mandatory fields, thus ensuring completeness of evaluation and higher detection rate, which in turn might explain higher incidence. The presence of CRAB features has long been suggested as poor prognostic markers, owing to the end-organ involvement and being manifestations of advanced disease.

Hypercalcemia was present in an extremely high proportion of our patients (60%) compared with those listed in the literature (11.3%-31%).^14,16,30,35-37^ We included all cases of symptomatic and asymptomatic hypercalcemia, which might also explain the differences in the overall incidence compared with other studies. The high incidence of hypercalcemia can be attributed to extensive bone disease and late presentation. The presence of hypercalcemia did not have a significant impact on OS in our study (Fig 3A), contrary to Nakaya et al^38^ (32 months *v* 101 months, *P* = .038).

Renal dysfunction, which was present in 60%, was strikingly much higher when compared with 18%-27.2% of patients mentioned in other studies.^14,16,35-37^ The high incidence of renal dysfunction can be again attributed to the late presentation of the patients. Also, this being a field study wherein all the patients were included in the database with no selection bias in choosing the patients, the results would represent the ideal percentages when compared with the randomized controlled trials or other observational studies. The presence of renal dysfunction at diagnosis had a significantly inferior OS at all time points in our study population (*P* = .04) (Fig 3B). The median OS in patients with and without renal disease in our study was 93.8 months *v* 139.89 months (*P* = .04), whereas Nayaka et al reported 101 months *v* 96 months (*P* = 0.98). The OS at 10 years in our study, in comparison with the study by Usmani et al,^39^ was 79% vis-à-vis 47%. In a study by Goswami et al,^40^ renal dysfunction had inferior survival (96 months *v* 64.5 months), but the results were not statistically significant. Another study by Gupta et al^21^ suggested a median OS of 81 weeks in NDMM with renal dysfunction.

Incidence of anemia in our study (74%) was comparable with other national and international studies variably reported between 49% and 100%.^14,16,30,35,36^ Anemia is the second commonest presentation across the world, as was also seen in our study. The presence of anemia at diagnosis showed a significant association with a shorter OS (*P* = 0.0039; Fig 3C), which was similar to the findings of other studies.^39,40^ On the contrary, Nakaya et al^38^ did not find any significant impact of anemia at diagnosis on overall outcomes (101 *v* 96 months, *P* = .858). The 10-year OS in patients with anemia in our study was 76.7% vis-à-vis 42% in a study by Usmani et al.^39^ The median OS in MM patients with anemia at diagnosis was found to be 93 weeks comparable with 141 weeks in a different Indian study.^21^ Anemia reflects the marrow burden of the disease and the aftermath of the proinflammatory cascade in the bone marrow niche because of myeloma and will be secondary to the renal failure. Although anemia is not included in the current revised International Staging System (R-ISS) staging, it remains to be a crucial prognostic factor as was found in our study.

The bone disease was the commonest CRAB feature in our study, with an incidence of 87%. The reported incidence in the literature varies from 29% to 96.9%.^14,16,30,35-37^ The bone disease in our study was detected not merely based on the skeletal survey, but with liberal usage of positron emission tomography scan or magnetic resonance imaging wherever felt necessary. Studies have already emphasized the increase in the incidence of bone disease by employing positron emission tomography at diagnosis, as seen in our study. The presence of the bone disease did not have any impact on survival, probably because of the skewed positive percentage in our population (Fig 3D). Although the bone disease is classically associated with increased morbidity, it is not associated with lower survival, as was also seen in our patients.^38,39^

Our study has increased higher ISS stage affliction compared with other published literature, which has a more homogenous distribution.^14,22,40-43^ As described in previous sections on CRAB features, the delayed presentation of the patients to the medical facilities can attribute to the higher ISS III patients. The comparison of OS among various ISS stages with the available literature is tabulated in Table 2. Although the difference in the estimated OS among the three stages was not statistically significant, the difference between ISS III stage and stages I and II was significant. This could have been possible because of the smaller sample size in stages I and II.

The number of patients with cytogenetic anomaly in our study was much smaller than that reported in the west because of lack of in-hospital facilities and outsourcing the tests and lack of plasma enrichment facilities in the country till a couple of years back. Also, as most of the patients were referred after initial evaluation at primary centers, the cytogenetic evaluation was missed by the primary physician because of lack of awareness, financial reasons, or nonavailability of universal cytogenetics facilities. This poor evaluation is a major limitation in interpreting the R-ISS results of our data.

Although LDH is a very cost-effective way of prognosticating MM and also a crucial part of R-ISS staging, the data on the LDH from our country are sparse. This is one of the larger study cohorts wherein LDH was prospectively evaluated. Raised LDH was seen in 57% of our study population, which was much higher compared with other published literature ranging from 10% to 15%.^21,22^ Mandatory LDH evaluation at diagnosis and late presentation might have attributed to the increased incidence. Raised LDH led to poorer survival, although the difference in survival between the groups with and without raised LDH was not significant (Fig 4B). The median OS in patients with raised LDH was reported to be 12-15.5 months in other studies.^21,44^ In a study by Usmani et al,^39^ raised LDH was significantly associated with a more inferior 10-year OS, but the same was not significant in a regression multivariate analysis.

We are among the first few to comment on the effect of co-morbidities on the OS of the patients with MM. Myeloma being a disease of the elderly, patients in this age group often suffer from other co-morbidities (eg, diabetes, hypertension, cardiovascular, renal, and respiratory diseases). Both the co-morbidities and the treatment for these co-morbidities can have severe impact or interactions with the natural biology of myeloma. Co-morbidities were present in 25% of our study population. The patients with co-morbidities did not have a significant impact on survival in our study, suggesting that disease control of co-morbidities can evade the adverse impact of these diseases on MM.

Our study followed up a large study population (n = 696) in real time using an online platform and hybrid application. The other major strength of the study is the multiethnic clientele in our study rather than studies emerging from tertiary care centers evaluating from only certain parts of the country. The median follow-up of our study was longer as compared with other Indian studies. Cytogenetics and β2 microglobulin were not available for a portion of the study group, impeding the calculation of ISS and R-ISS in the entire population. Extramedullary disease at presentation can have adverse impact on the overall outcomes of these patients, which was not evaluated in this study.^45^ We have not dwelled into the impact of the therapy details as the treatment was heterogenous in our clientele, and also in a relapsing-remitting disease that ought to receive multiple lines of therapy, impact of a singular therapy is not relevant. Although minimal residual disease status and transplant have an impact on the OS, we have not reported as it was out of scope of the current study.^4,46^ We have not reported the progression-free survival in our study, owing to the difficulty in the timely evaluation of the pan-India population at predecided intervals.

In conclusion, our study showed that the presence of anemia, weight loss, and renal dysfunction at diagnosis led to the significantly poorer OS in patients with MM. We had more patients belonging to advanced ISS stages. The real-world data show OS comparable with the published western literature. The use of such hybrid applications can aid in better data-keeping in resource-constrained settings.

## Figures and Tables

**TABLE A1:**
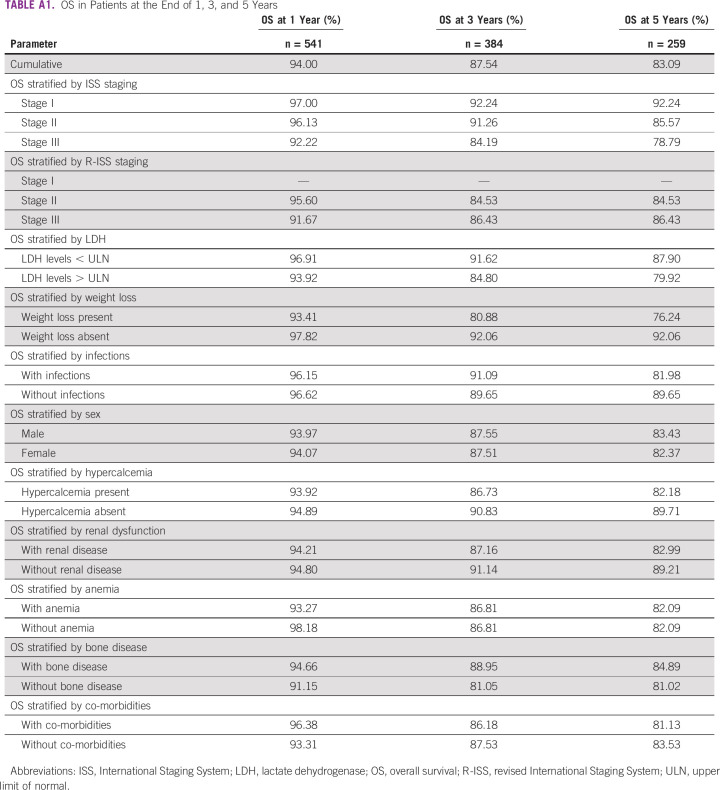
OS in Patients at the End of 1, 3, and 5 Years

**TABLE A2:**
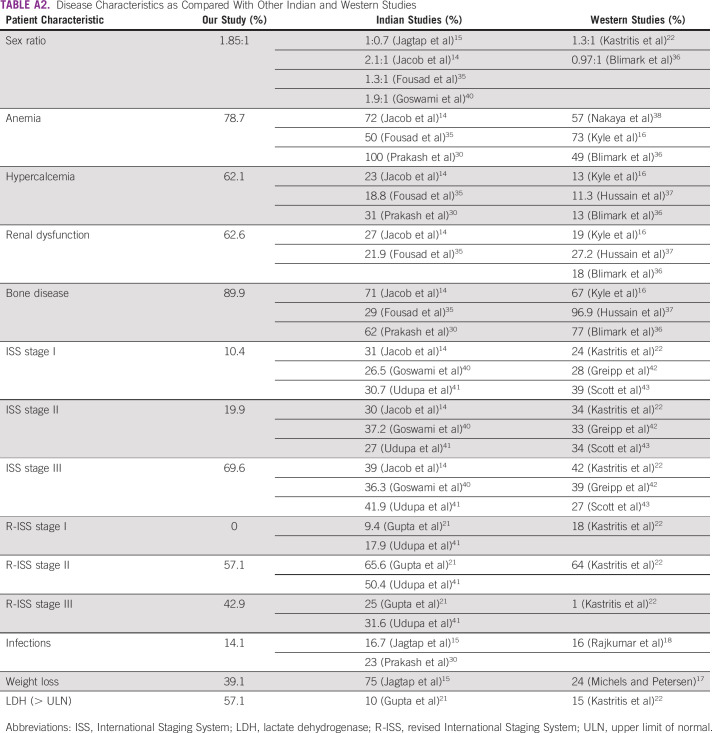
Disease Characteristics as Compared With Other Indian and Western Studies
